# Cross-Modal Interaction Between Perception and Vision of Grasping a Slanted Handrail to Reproduce the Sensation of Walking on a Slope in Virtual Reality †

**DOI:** 10.3390/s25030938

**Published:** 2025-02-04

**Authors:** Yuto Ohashi, Monica Perusquía-Hernández, Kiyoshi Kiyokawa, Nobuchika Sakata

**Affiliations:** 1 Graduate School of Science and Technology, Nara Institute of Science and Technology, Ikoma 630-0192, Japan; perusquia@ieee.org (M.P.-H.); kiyo@is.naist.jp (K.K.); 2 Faculty of Advanced Science and Technology, Ryukoku University, Otsu 520-2194, Japan; sakata@is.naist.jp

**Keywords:** virtual reality, cross-modal, haptics

## Abstract

Numerous studies have previously explored the perception of horizontal movements. This includes research on Redirected Walking (RDW). However, the challenge of replicating the sensation of vertical movement has remained a recurring theme. Many conventional methods rely on physically mimicking steps or slopes, which can be hazardous and induce fear. This is especially true when head-mounted displays (HMDs) obstruct the user’s field of vision. Our primary objective was to reproduce the sensation of ascending a slope while traversing a flat surface. This effect is achieved by giving the users the haptic sensation of gripping a tilted handrail similar to those commonly found on ramps or escalators. To achieve this, we developed a walker-type handrail device capable of tilting across a wide range of angles. We induced a cross-modal effect to enhance the perception of walking up a slope. This was achieved by combining haptic feedback from the hardware with an HMD-driven visual simulation of an upward-sloping scene. The results indicated that the condition with tactile presentation significantly alleviated fear and enhanced the sensation of walking uphill compared to the condition without tactile presentation.

## 1. Introduction

In recent years, incorporating physical elements into Virtual Reality (VR) environments has been deemed essential in VR research [1,2]. As part of this effort, numerous studies have focused on horizontal movement techniques in virtual environments, such as Walking in Place (WIP) and RDW [3,4,5,6]. However, replicating vertical movements, such as slopes and stairs, has proven to be challenging. The primary challenges include the fear of falling when using physical inclines while wearing HMDs. As a result, VR experiences involving vertical movement have remained difficult to implement [7,8]. If vertical movement can be replicated in a virtual environment, it would enable the creation of three-dimensional virtual spaces in a limited area, potentially enhancing the VR experience. Furthermore, stairs and slopes are common in the real world, making research on replicating vertical movement in VR essential.

As an example of research on vertical movement in virtual environments, Hoshi et al. recreated the sensation of climbing a slope by physically tilting a treadmill [8]. They successfully recreated the sensation of walking on a steep slope in a virtual environment by tilting the treadmill. However, despite displaying the walkable range with a wireframe and taking precautions to prevent stepping out, users still experienced fear while walking. Additionally, Dominik et al. recreated the sensation of stepping over a step in a virtual environment by attaching an actuator that operates vertically to the sole of the shoe [7]. However, issues such as instability and difficulty walking arose due to the height added by the actuator attached to the shoe’s sole. These studies employed physical methods to create and perceive slopes and steps. The generated slopes and steps were real and, therefore, highly reproducible. However, they were dangerous and instilled fear in users due to their physicality. Consequently, many studies have explored using cross-modal effects [9]. The cross-modal effect refers to a phenomenon in which multiple senses are stimulated simultaneously, influencing each other and altering perception based on the stimuli received from different senses. Human senses are interrelated, and research has shown that haptic sensations can be elicited through visual stimuli. Furthermore, there is a strong interaction between visual and haptic senses [10].

**Figure 1:**
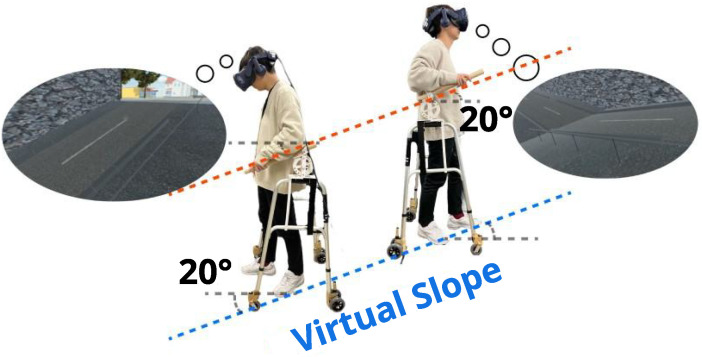
The virtual slope is represented by the physical grasp angle, and a slanted handrail induces the sensation of walking on a slope.

Hence, we propose using the haptic sensation of gripping a sloped handrail, such as those attached to slopes or escalators, as a means to induce a cross-modal effect. In our proposed method, we enhance the perception of walking on a slope by simultaneously providing haptic feedback through the walker-type handrail device (Figure 1) and visual stimulation of a slope image displayed on an HMD. This simultaneous presentation of haptic and visual stimuli creates a cross-modal phenomenon that intensifies the perception of walking on a slope. In our proposed method [11], a handrail device that can move along a plane with the walker is provided, allowing the user to experience the walking scene displayed on the HMD. Additionally, by implementing an adjustable angle function on the proposed handrail device, it is possible to simulate the sensation of continuously ascending or descending a slope.

When using this handrail device, users experience no fear of falling, as they are walking on a flat surface. Thus, it is possible to safely simulate the sensation of walking on a slope without adverse side effects. This study aims to propose and implement a method for simulating the sensation of walking on a slope in a virtual environment without inducing fear. The contributions of this study are outlined as follows.

- We enhanced the sensation of walking on a slope by integrating visual and haptic information.
- We simulated the sensation of walking on a slope on a flat surface without employing any physical incline.
- We simulated the sensation of walking on a slope without impeding natural walking, achieved simply by grasping a handrail, requiring no complex training.
- We simulated the sensation of walking on a slope while minimizing the feeling of fear.

## 2. Related Work

### 2.1. Cross-Modality

Cross-modal interactions have become a prominent focus in VR research. Narumi et al. developed a system that induces the sensation of walking straight ahead, even when users are actually walking along a curved wall. This illusion leverages the interaction between vision and touch [5]. This study enabled unrestricted continuous walking within a small space in a virtual environment. Steinicke et al. demonstrated that by manipulating only visual information, users perceive themselves as walking in a straight line rather than a curve once a certain threshold is exceeded [3,4]. Cross-modality involves the combination of multiple senses, and research has explored the integration of taste and vision. It has been confirmed that integrating taste and visual information can alter taste perception [12,13]. Additionally, studies have investigated the combination of auditory and visual information, and its effectiveness has been demonstrated [14]. Additionally, Kruijff et al. enhanced immersion by combining foot haptic feedback, auditory, and visual stimuli while participants were seated. This demonstrated the effectiveness of multi-sensory integration [15].

In this study, we propose using handrails to enhance the walking experience. Accordingly, we considered the findings on tactile presentation to the hand to be meaningful. Recent research has demonstrated that objects in both real and virtual environments can be perceived in different sizes. This finding has enabled the manipulation of grasp perception through visual interventions [16]. This study aimed to reconcile the disparities between visual information and grasp perception by using visual cues. Notably, the research successfully replicated multiple virtual objects using a single real-world object. Although this study explored the fusion of vision and grasp perception, it is essential to acknowledge a gap in the existing research landscape. Specifically, no prior studies have investigated the relationship between the angle at which a stick is grasped and its corresponding visual representation. This uncharted territory presents an intriguing opportunity for future research, potentially shedding light on unexplored aspects of human perception and interaction within virtual environments. Matsumoto et al. manipulated perception in a virtual environment through curvature manipulation and Redirected Walking [17]. Notably, they succeeded in making a square table in the real environment feel like a pentagonal table in the virtual environment. Extensive research has been conducted on the haptic manipulation of objects in virtual environments. Additionally, various methods for haptic presentation have also been studied [18,19]. Huang et al. developed the ‘Haptic-go-round’ platform, which enables haptic feedback from all directions to be synchronized with a virtual environment within a circular device. Wilberz et al. enhanced the sense of presence in the virtual environment by combining various facial haptic feedback methods with visual information [20]. Cheng et al. developed TurkDeck, a system that delivers haptic feedback in sync with the virtual world by having humans manipulate physical objects [21]. Thus, wearing specialized shoes can provide various forms of haptic feedback, enhancing immersion in the virtual environment.

### 2.2. Experience of Vertical Movement

There are several techniques in the study of horizontal movement, with RDW being the most prominent. One technique within RDW is known as the reset technique. Many studies using the reset technique involve changing the user’s direction while the virtual world is paused or redirected at specific points. A position with a high degree of movement freedom is calculated, and after guiding the user to this point, they are reset to it as the initial position, thereby reducing the impact of obstacles [22]. Additionally, unlike methods that apply redirection when the user is focused on a specific object, some techniques redirect users while their attention is diverted away from the object [23,24].

In contrast to research on horizontal movements, studies on vertical movements have not progressed as rapidly. To address this, Matsumoto et al. investigated vertical movements using drones [25]. Iwata et al. developed the Gait Master, a device that detects a walker’s movement using a position sensor and moves in the opposite direction of travel to counteract walking [26]. While this enabled the reproduction of steps, it also presented challenges, such as operational delay and the need for user habituation. Additionally, Schmidt et al. attempted to reproduce stepping motions on a flat surface using a leg-mounted device [7]. However, similar to Iwata et al.’s research [26], habituation was required. Additionally, difficulty in achieving natural flat walking was identified as a limitation. Hirao et al. attempted to replicate a sloping surface effect in on-the-spot walking using pseudo-haptic feedback [27]. Matsuda et al. provided a sense of walking in a virtual environment by delivering foot haptic feedback [28]. Narumi et al. replicated the sensation of climbing stairs in a virtual environment using cross-modal effects [9]. They presented simultaneous visual and haptic feedback to replicate the sensation of ascending and descending stairs without physical steps. Specifically, they replicated the edge of the stairs by placing small protrusions on the ground, allowing subjects to feel the sensation of ascending and descending stairs, even while walking on a flat surface. Nordahl et al. demonstrated that presenting vibratory haptic feedback from a shoe-type device while riding an elevator in a virtual environment can induce an illusion of vertical movement and height [29,30]. Rudolph et al. developed an omnidirectional treadmill for movement in large-scale virtual environments [31]. As a result, it became possible to simulate large-scale virtual environments even within confined spaces. Research aimed at reproducing physical steps has shown that large-scale equipment is required, habituation is necessary, natural walking is complex, and safety issues are present. Given these challenges, it is valuable to propose a method that can safely provide the sensation of walking on a slope in a virtual environment, without inducing fear, without requiring habituation, and allowing for natural walking.

### 2.3. Cross-Modal Interactions Between Visual Stimuli and the Sensation of Gripping a Handrail

The purpose of the proposed method is to safely evoke the sensation of walking on a slope without relying on complex devices or physical inclines. The user’s spatial cognition changes when grasping an inclined handrail. Unlike studies that use cross-modal phenomena through haptic stimulation of the feet and visual stimulation via HMDs, we utilize haptic feedback from the hands, which have a higher density of nerve cells than the feet. Therefore, we believe that this method has a high potential for reproducing the sensation of walking on a slope. The RDW method, which utilizes cross-modal interaction between vision and haptic sensations [5], is also applicable to vertical viewpoint manipulation [9]. Nordahl et al. demonstrated that adding vibratory haptic feedback enhances the illusion of self-motion in the vertical direction [30]. Based on these findings, we hypothesize that our proposed method could evoke the sensation of walking on a slope. In our proposed method, users can easily experience walking on a flat surface while holding onto a handrail, without the need for complex training. We believe this method could be applied to creating vast virtual worlds and construction site simulations by increasing the degree of freedom in vertical movement.

## 3. User Evaluations

### 3.1. Preliminary Experiment

Studies have shown that accurate angle estimation is not possible when perceiving the angle of a rod without visual information [32]. Based on this, it may be possible to influence the human perception of slope inclination by adding visual manipulation to the ambiguous perception of handrail inclination. Therefore, prior to the main experiment, we conducted a preliminary experiment to investigate the ambiguity in human perception of handrail inclination based solely on touch. The participants were blindfolded and instructed to provide verbal responses regarding the inclination of the handrail. The pilot study suggested that handrail inclination angles between 15 and 20 degrees could be estimated with less individual variation compared to other intervals when the handrail was grasped accurately. Additionally, relative angles could be estimated with reasonable accuracy when the change in inclination was 10 degrees or less. Therefore, we set the handrail inclination to 15 and 20 degrees.

### 3.2. User Study of Reproducing the Sensation of Walking on a Slope

**Figure 2:**
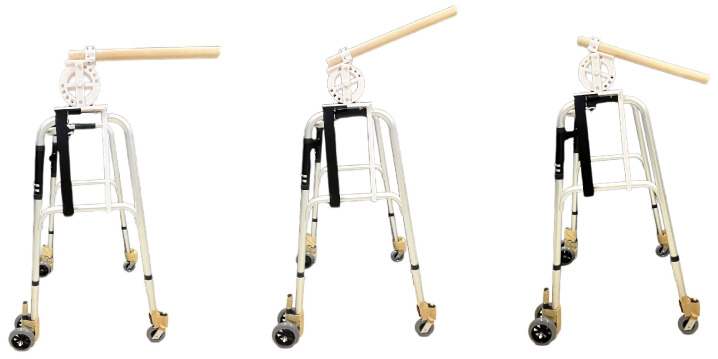
The walker-type handrail devices.

**Figure 3:**
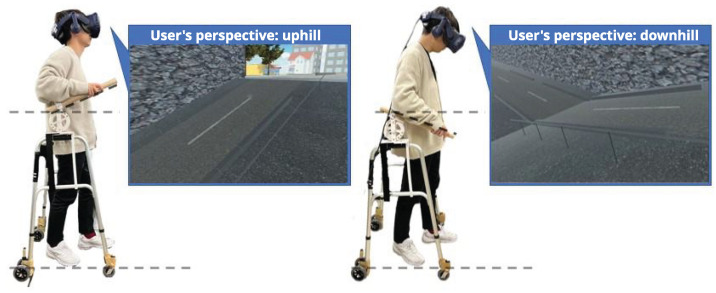
Overview diagram of the proposed methodology.

In this experiment, we reproduced and evaluated the sensation of walking on a slope using a cross-modal phenomenon, combining haptic stimulation from a walker-type handrail device (Figure 2) and visual stimulation from HMDs, as shown in Figure 3.

#### 3.2.1. Participants

We recruited 25 participants (aged 20–24, mean age 22.4), including 9 females and 16 males. All participants reported previous experience with HMDs. All participants had normal or corrected-to-normal vision. All participants provided informed consent before the study, and the experimental procedure was approved by IRB number 2022-54.

#### 3.2.2. Experiment Design

**Figure 4:**
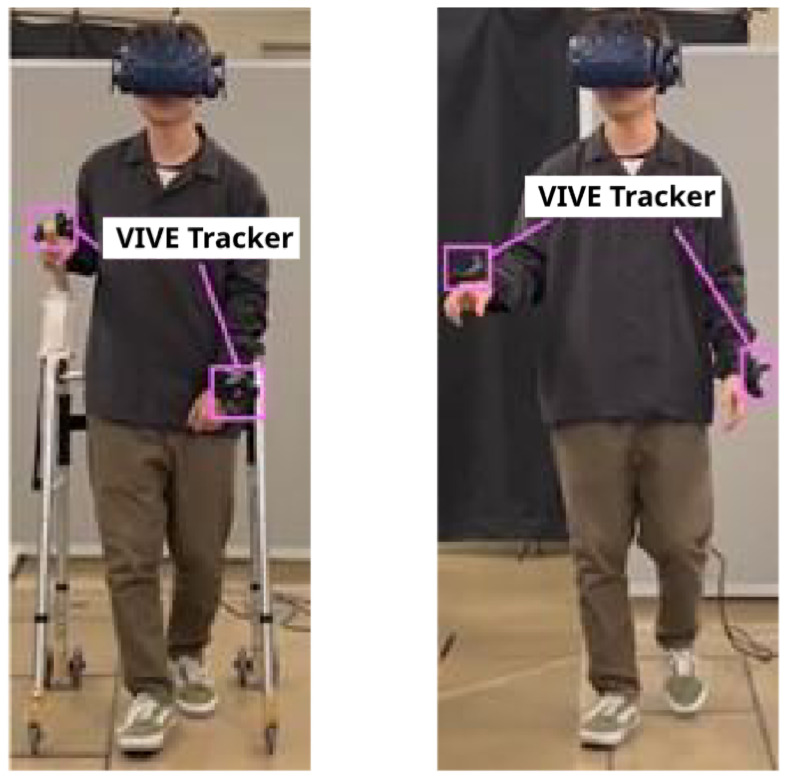
Experiment: The left side represents the tactile condition, while the right side represents the non-tactile condition.

The experiment employed a 2×2 within-subjects design. There were two haptic conditions (haptic and non-haptic) and two angle conditions (20 and 15 degrees) (Figure 4). The order of conditions was randomized.

In the haptic condition, participants walked while holding the handrail device; in the non-haptic condition, they walked without holding anything. The ground remained level in both conditions.

#### 3.2.3. Hardware

In the experiment, participants walked straight from the starting point while wearing HMDs and holding the walker-type handrail device. The walking path measured 2 m in width and 5 m in length. Participants then turned around and returned to the starting point.

The entire walking area met the requirements of the VIVE room-scale play area. Additionally, there were no obstacles or impediments in the play area, and the ground was evenly leveled. The walker-type handrail device used in the experiment is shown in Figure 2. The experiment was conducted with participants holding the handrail attached to the walker. This method was chosen because a walker does not hinder walking, even when held. The walker used was a commercially available model for nursing care (LEJIEYIN, Model B, width 53 cm × height 76.5 cm × depth 46 cm, weight 4.2 kg). It has four legs and is designed to remain stable even when weight is applied to the handrail. The tips of the four legs are equipped with 360-degree rotating casters. However, the casters were fixed to prevent rotation, as the experiment was designed to investigate movement only in a straight direction.

**Figure 5:**
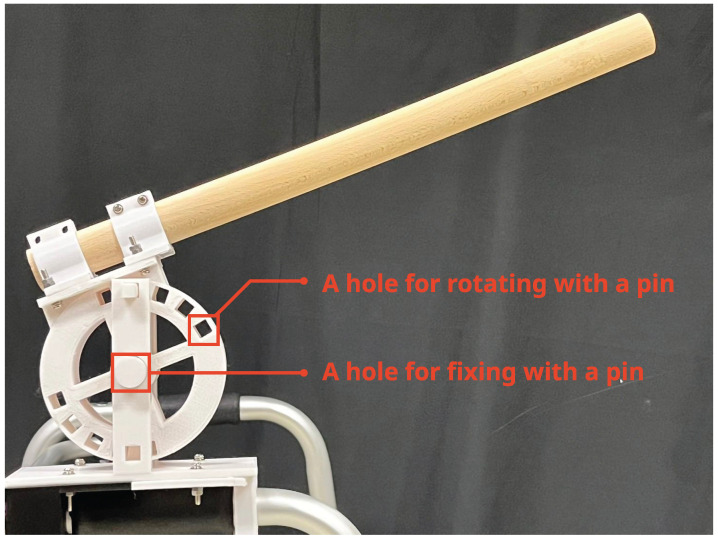
Rotational mechanism made by 3D printer.

A rotating mechanism was attached to the walker (Figure 5). The rotating mechanism was fabricated using a 3D printer (Anycubic Mega X, PLA 1.75 ‘mm filament). The rotating mechanism included a central hole for rotation and additional holes along the circumference to fix the rotation angle. The rotation angle could be fixed by inserting cylindrical or rectangular pins into these holes. The rotation angle could be adjusted from 0 to 50 degrees in 5-degree increments. The total weight of the walker-type handrail device combined with the rotating mechanism was 4.3 kg.

The VIVE Pro EYE was used as the HMD in the experiment. The VIVE Pro EYE has a resolution of 1440 × 1600 pixels per eye, a refresh rate of 90 Hz, and a maximum field of view of 110°. The HMD and VIVE Tracker are tracked by HTC/Valve’s Lighthouse system. An Intel computer with a Core i5 processor, 16 GB of memory, and a GeForce RTX 3060 GPU was used to render the virtual environment. The virtual environment was rendered in Unity 3D on Windows 10.

#### 3.2.4. Measurements

A pre-experiment questionnaire gathered participant information (age, gender, VR experience, visual acuity, and dominant hand), and a Simulator Sickness Questionnaire (SSQ) [33] consisting of 16 questions was used to assess simulator sickness. The SSQ score was calculated by multiplying the total of all items by 3.74 on a 4-point Likert scale [0 (not applicable) to 3 (severely applicable)]. The Japanese version of the Igroup Presence Questionnaire (IPQ) assessed the participants’ sense of presence. The IPQ consists of four subscales (Pres: General Presence, SP: Spatial Presence, Inv: Involvement, Real: Experienced Realism) and is rated on a 5-point Likert scale. Pres, SP, Inv, and Real consist of one, five, four, and five items, respectively. The IPQ scores were calculated based on Schubert et al. [34]. A questionnaire was developed to measure the sensation of walking on a slope (hereinafter referred to as Slope Questionnaire). The slope walking sensation questionnaire included the following items.

- Did you feel like you were walking on a slope during the VR experience?
- How often did you feel like you were walking on a slope during the VR experience?
- Did you feel that the movements in the virtual environment matched the movements in the real world during the VR experience?
- How often did you feel that your movements in the virtual environment matched your movements in reality during the VR experience?
- How much fear did you feel?

The five questions mentioned above were answered separately for the ‘looking downslope’ and ‘looking upslope’ sections using a 7-point Likert scale, ranging from 1 to 7. According to a study by Norman and Geoff [35], values above four on the Likert scale obtained in VR experiences can be statistically processed parametrically. However, in a study by Matsumoto et al. [5], similar values obtained from question items were averaged and analyzed non-parametrically for an evaluation. In this experiment, the evaluation method used by Matsumoto et al. was adopted.

**Figure 6:**
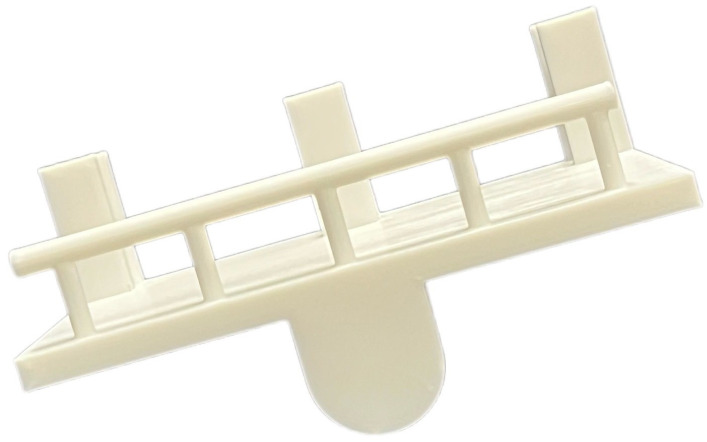
Self-report slope angle measuring device. Participants can answer the self-report perception of slope angle felt by the participants as a number via the Inertial Measurement Unit (IMU) of a smartphone application for iPhone 14.

The slope angle measuring instrument shown in Figure 6 was used to assess the inclination angle. This instrument has a width of 10 cm and a height of 15 cm and represents a reduced slope model in the virtual environment used in this experiment. After each experimental trial, participants were instructed to reproduce the perceived ground inclination by adjusting the slope-measuring instrument. In a preliminary questionnaire, some participants noted in the free comment section that it was difficult to quantify the angle because they were unaware of the slope’s angle. Therefore, we believe that using this self-report slope angle measuring instrument allows participants to judge the slope’s inclination based on their intuition.

#### 3.2.5. Procedure

Participants were guided to the starting point in the experiment and instructed to walk straight to the turning point. They were then instructed to walk straight back to the starting point from the turning point. In the haptic condition, participants were instructed to hold the walker-type handrail device. The total duration of the experiment, including instructions and questionnaire completion, was 40 minutes. Before the experiment, all participants signed an informed consent form after receiving an explanation of the experiment.

Additionally, participants completed the SSQ both before and after the experiment. Participants grasped a handrail set at zero degrees while viewing a flat slope scene to familiarize themselves with the hand avatar and experimental scenery. The slope scene used in this experiment was modeled after an underground tunnel where a railway and a road intersect. This scene was chosen so that participants could estimate the slope based on their perception rather than external cues, such as the inclination of trees, as the tunnel walls obscured such external factors.

In all trials, participants were instructed to ascend the slope from the starting point to the turning point. They were then instructed to descend the slope from the turning point back to the starting point. After each trial, participants responded to a questionnaire about the sensation of walking uphill and to IPQ and slope angle measurements to measure their perception of walking uphill, without the VR headset.

## 4. Results

We analyzed the questionnaire results from twenty-seven participants, as well as the inclination angle evaluations obtained using a slope-measuring instrument. Furthermore, there were no signs of participants falling, stumbling, or being hindered from walking naturally during the experiment.

**Figure 7:**
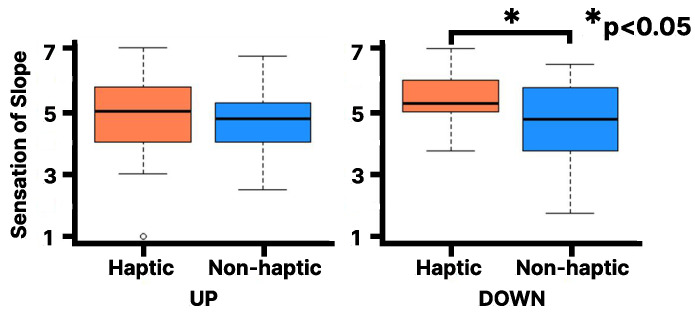
Evaluation of Slope Questionnaire on the virtual 15 degrees slope: Left for UP, Right for DOWN [1 (strongly disagree) to 7 (strongly agree)].

**Figure 8:**
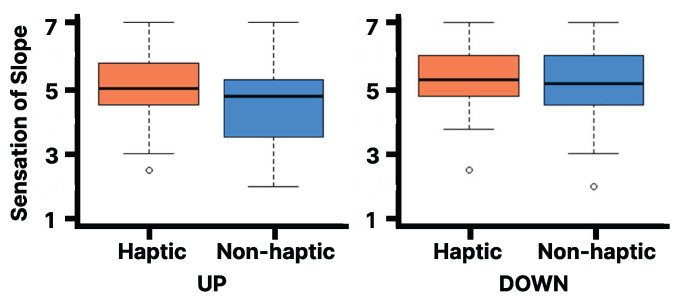
Evaluation of Slope Questionnaire on the virtual 20 degrees slope: Left for UP, Right for DOWN [1 (strongly disagree) to 7 (strongly agree)].

As shown in Figure 7, the Wilcoxon signed-rank test on the Slope Questionnaire results reveal that the median rank (Mdn = 5.25) for the downhill haptic condition at 15 degrees of visual stimulation is statistically significantly higher than for the non-haptic condition (Mdn = 4.75, Z = 222, *p* < 0.0009, r = 0.15). Note that *p* refers to the *p*-value, Z to the Z score, Mdn to the median, and r is the effect size. The lines in the box plot indicate the median value and the interquartile range. However, no statistically significant difference was observed between the haptic and non-haptic conditions in the uphill condition. Additionally, no statistically significant difference was observed between the haptic and non-haptic conditions when the visual stimulus from the slope image was at 20 degrees, even when analyzed separately for uphill and downhill (Figure 8).

**Figure 9:**
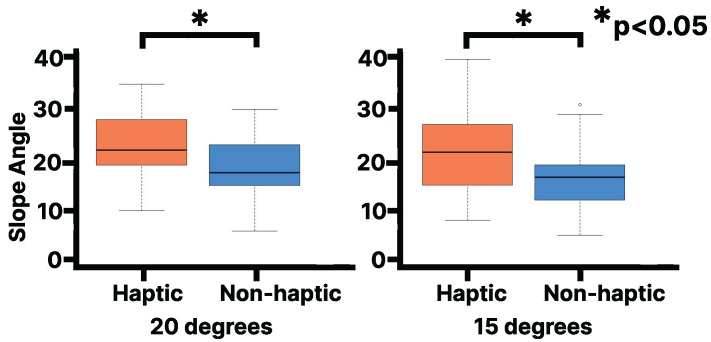
Evaluation of slope angle using the self-report slope angle measuring device on the virtual 20 and 15 degrees slope. The vertical axis represents the angle at which subjects responded to the instrument.

Next, we discuss the results of participants’ self-report evaluation of the slope angle using the measuring instrument. When the visual stimulus from the slope image was set to 15 degrees, the median rank for the haptic condition (Mdn = 21.5) was statistically significantly higher than that for the non-haptic condition (Mdn = 16.5, Z = 273.5, *p* < 0.0001, r = 0.92). When the visual stimulus from the slope image was set to 20 degrees, the median rank for the haptic condition (Mdn = 22.0) was higher than that for the non-haptic condition (Mdn = 17.5, Z = 225.5, *p* < 0.006, r = 0.60; Figure 9).

**Figure 10:**
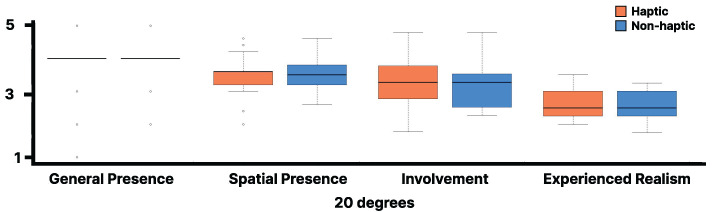
IPQ results for the virtual 20 degrees slope.

**Figure 11:**
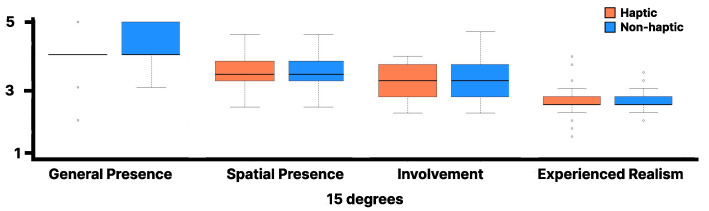
IPQ results for the virtual 15 degrees slope.

The Wilcoxon signed-rank test was performed between the haptic and non-haptic conditions for each item of the IPQ with visual stimulation at 20 degrees, but no significant differences were found for any item (Figure 10). The lines in the box plot indicate the median value and the interquartile range. Similarly, the Wilcoxon signed-rank test was conducted between the haptic and non-haptic conditions for each item of the IPQ with visual stimulation at 15 degrees, and no significant differences were found for any item (Figure 11). The lines in the box plot indicate the median value and the interquartile range.

**Figure 12:**
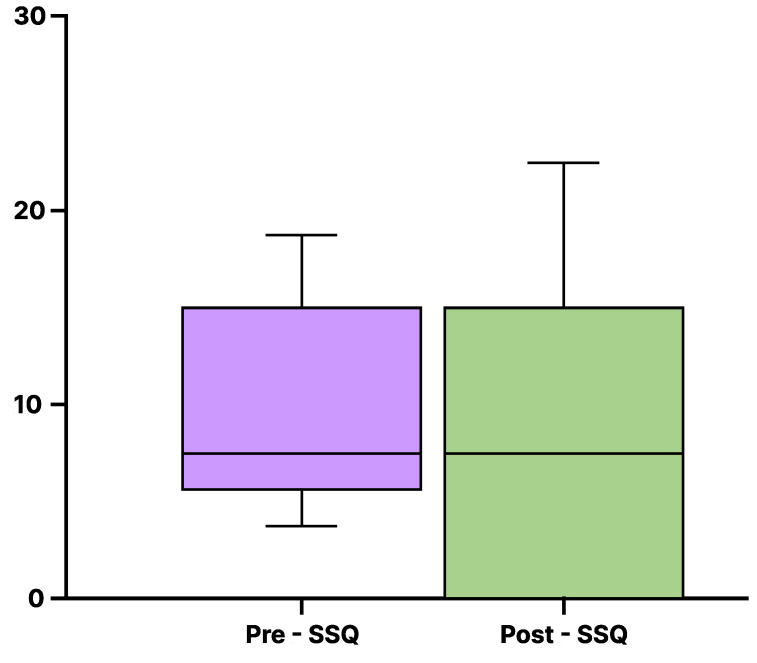
SSQ total score.

For the SSQ, as shown in Figure 12, the score before experiencing the proposed system (Mdn = 7.77) was nearly identical to the score after experiencing the system (Mdn = 9.86). The lines in the box plot indicate the median value and the interquartile range. Additionally, the Wilcoxon signed-rank test showed no significant difference between the pre- and post-experience scores for the proposed system (*p* = 0.69, r = 0.10).

**Figure 13:**
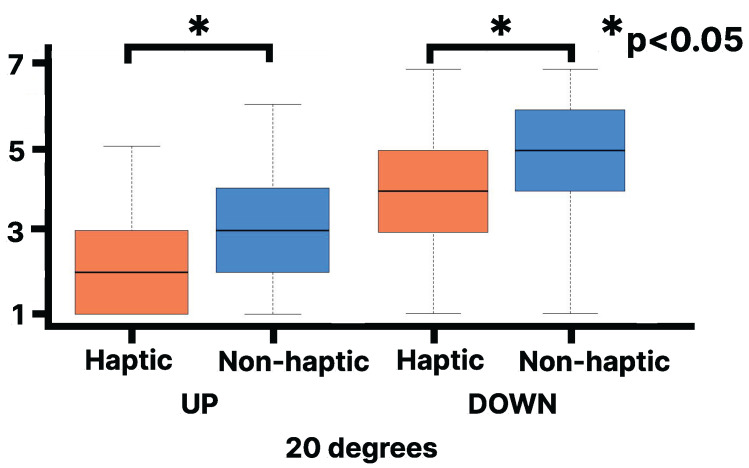
Evaluation of fear for walking up and down/haptic and non-haptic conditions on the virtual 20 degrees slope [1 (strongly disagree) to 7 (strongly agree)].

Figure 13 compares the qualitative fear ratings in the haptic and non-haptic conditions when walking up and down a virtual slope. The lines in the box plot indicate the median value and the interquartile range. The Wilcoxon signed-rank test indicated that the median rank for the haptic condition during a 20-degree incline ascent (Mdn = 2) was statistically significantly lower than that for the non-haptic condition (Mdn = 3, Z = 4, *p* < 0.0005, r = 0.75). The Wilcoxon signed-rank test indicated that the median rank for the haptic condition during a 20-degree slope descent (Mdn = 4) was statistically significantly lower than that for the non-haptic condition (Mdn = 5, Z = 32.5, *p* < 0.02, r = 0.45). The Wilcoxon signed-rank test indicated that the median rank for the haptic condition during a 15-degree uphill slope (Mdn = 4) was statistically significantly lower than that for the non-haptic condition (Mdn = 5, Z = 32.5, *p* < 0.003, r = 0.5). Fear ratings were also statistically significantly higher for the downhill conditions compared to the uphill conditions across all tests.

## 5. Discussion

In the evaluation, the Slope Questionnaire revealed a significant difference between the haptic and non-haptic conditions for a downhill slope with 15 degrees of visual stimulation. However, no statistically significant difference was found between the haptic and non-haptic conditions for the 20-degree uphill and downhill slopes and the 15-degree uphill slope with visual stimulation. Additionally, regardless of the presence of the haptic condition, fear was higher on downhill slopes than on uphill slopes. This suggests that visual stimulation with images of downhill slopes induces fear of slopes. The lack of a significant difference between haptic and non-haptic conditions with 20-degree visual stimulation may be due to foot discomfort. According to Ishikawa et al. [8], three types of changes in foot force occur on level ground and slopes, with significant changes in ankle angle, especially on steep slopes. Thus, the mismatch between ankle angle and somatosensory perception on steep slopes may hinder the improvement of gait perception. The significant difference in slope perception between the haptic and non-haptic conditions on a downhill slope with a 15-degree visual stimulus may be attributed to the absence of a mismatch between ankle angle and somatosensory perception, potentially arising from the interaction of visual and other stimuli. We expect that the interrelationship between the ankle angle, somatosensory perception, and slope walking sensation will be further elucidated by conducting experiments on slopes with a more moderate gradient.

Significant differences were found between the haptic and non-haptic conditions in the quantitative evaluation of slope angle using the measuring device, suggesting that walking on a slope while holding a handrail in a virtual environment may amplify the perception of the slope angle. In particular, the 15-degree slope amplified the perception of walking on a slope more than the 20-degree slope. This suggests that the interaction between visual and hand haptic perception may be stronger than that between visual and foot haptic perception on slopes with lower inclination angles.

Next, a detailed analysis of the quantitative evaluation of slope angles using the measuring device, separated by male and female subjects, revealed significant differences in the results for males only, while no significant differences were observed for females. This is most likely because the number of female subjects was smaller than that of male subjects. Additionally, a study by Luc et al. investigated the differences in self-orientation between males and females, reporting that females tended to rely more on visual information [36]. This may have led to higher angle evaluations by the measuring device for males, who relied more on haptic information than females. These comments suggest that the cross-modal interaction between visual and haptic senses enhances the sensation of walking on a slope.

**Figure 14:**
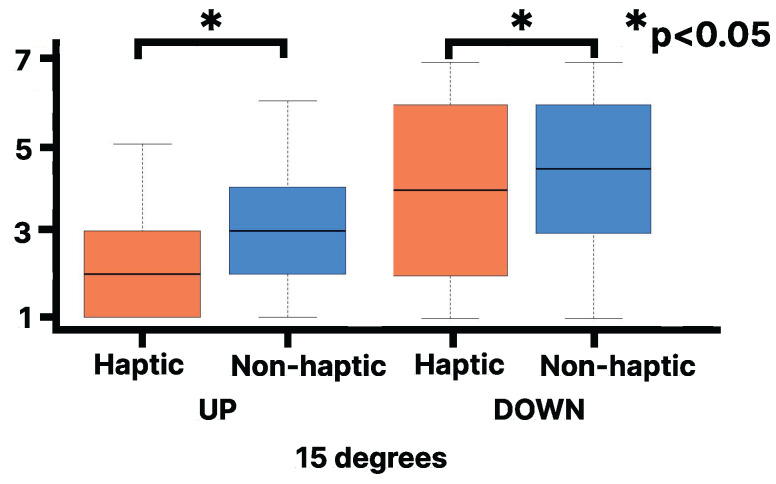
Evaluation of fear for walking up and down/haptic and non-haptic conditions on the virtual 15 degrees slope [1 (strongly disagree) to 7 (strongly agree)].

Using the walker-type handrail device reduced the fear associated with virtual downhill walking on a slope. The fear scores suggest that virtual upslope and downslope walking with the walker-type handrail device did not produce sufficient fear to interfere with walking. Additionally, as shown in Figure 13 and Figure 14, the fear score significantly decreased in the haptic condition compared to the non-haptic condition, and using the walker-type handrail device enhanced the sensation of walking uphill in the haptic condition. These results suggest that this device has the potential to provide users with a safe sensation of walking on a hill. No comments were received regarding the difficulty in walking or the need to adapt to using the walker-type handrail device. This suggests that the device can provide a natural sensation of walking on a slope without requiring adaptation.

As shown in Figure 10 and Figure 11, no significant differences were observed in the IPQ scores between the haptic and non-haptic conditions, suggesting that the sense of realism was not impaired in either condition. This suggests that using the walker handrail device enhances immersion in the virtual environment without diminishing the sense of presence. Furthermore, the absence of significant differences in SSQ scores suggests that the proposed method can be experienced without inducing VR sickness.

To ensure that participants remained unaware of using the handrail during the experiment, it was necessary not to reveal its presence to them beforehand. Since this aspect of the experiment design cannot be altered at this stage, our approach remains consistent across all conditions. Therefore, knowledge of the real-world handrail’s shape does not pose a problem. That said, further investigation is needed to evaluate the effects of grasping the handrail, both with and without participants’ awareness.

### Future Work

Based on the results of a pilot study, the handrail angles were set to 15 degrees and 20 degrees. Therefore, handrails with other angles were not used in the experiment. Future research should explore other angles. The current walker-type handrail device requires users to walk while holding the handrail, making it difficult to rely on it in the same way as a real handrail.

After the experiment, participant No. 4 (male, age 22) commented, ‘It is impossible to grasp it like a real handrail.’ Participants had to walk while holding the handrail and guiding the walker-type handrail device. As a result, it was difficult to re-grip the handrail, which decreased satisfaction with the handrail’s realism. On the other hand, although the current walker-type handrail device is used differently from an actual handrail, it was still able to provide the sensation of climbing a slope. If a walker-type handrail device that can be used like an actual handrail is developed, we can expect to achieve greater realism in the sensation of climbing a slope and further amplify the sense of walking on a slope.

We are currently developing a device that replicates a real handrail through motor control. By controlling the rotation of the walker’s wheels and the grasping component, the mechanism allows the grasping part to move in sync with the user’s walking. This device can faithfully replicate real handrail grasping methods and is expected to be even more effective.

## 6. Conclusions

In this paper, we propose a method to enhance the sensation of walking on a slope in a virtual environment using handrails. We leveraged the real-world use of handrails for vertical movement on stairs and slopes, presenting haptic stimulation when grasping the handrail as a cross-modal factor. Our user study confirmed that the visual information of slopes and handrails presented by the HMD, combined with haptic information from the handrails, induces cross-modal interaction.

In the experiment, participants were instructed to walk straight on a flat surface while holding the walker-type handrail device. We used both qualitative evaluations (Slope Questionnaire) and quantitative evaluations (a slope angle measuring instrument) to assess the experiment. In the evaluation, the sensation of walking on a slope was significantly enhanced in the haptic condition compared to the non-haptic condition at 15 degrees downhill. In the behavioral evaluation, the sensation of walking on a slope was significantly enhanced in the haptic condition compared to the non-haptic condition at both 20 and 15 degrees. Similarly, fear scores were significantly lower in haptic conditions compared to non-haptic conditions. This indicates that using the walker-type handrail device can reduce fear. Additionally, the participants’ walking was not hindered during the experiment. Using handrails amplified the sensation of walking on a slope on a flat surface without impairing immersion in the virtual environment. The evaluation results indicate that amplifying the sensation of walking on a slope through cross-modal interaction between vision and passive haptics is effective.

With the current device, it is challenging to apply it in directions other than straight ahead, but rotation is possible by removing the implemented restrictions on the handrail. For example, we believe it is possible to reproduce vast maps in limited spaces by combining the research of Matsumoto et al. [5] with our proposed method. It can also be applied to rehabilitation for people with difficulty ascending and descending slopes.

## Data Availability

The data presented in this study are available on request from the corresponding author.
